# Dominant Antiviral CD8^+^ T Cell Responses Empower Prophylactic Antibody-Eliciting Vaccines Against Cytomegalovirus

**DOI:** 10.3389/fimmu.2022.680559

**Published:** 2022-01-27

**Authors:** Iris N. Pardieck, Suzanne van Duikeren, Dominique M. B. Veerkamp, Dena J. Brasem, Anke Redeker, Jeroen van Bergen, Wanda Han, Ferry Ossendorp, Gerben Zondag, Ramon Arens

**Affiliations:** ^1^Department of Immunology, Leiden University Medical Center, Leiden, Netherlands; ^2^Immunetune BV, Leiden, Netherlands

**Keywords:** cytomegalovirus, prophylactic vaccination, DNA vaccination, antibody response, synthetic long peptides, T cells

## Abstract

Human cytomegalovirus (HCMV) is an ubiquitous herpesvirus that can cause serious morbidity and mortality in immunocompromised or immune-immature individuals. A vaccine that induces immunity to CMV in these target populations is therefore highly needed. Previous attempts to generate efficacious CMV vaccines primarily focused on the induction of humoral immunity by eliciting neutralizing antibodies. Current insights encourage that a protective immune response to HCMV might benefit from the induction of virus-specific T cells. Whether addition of antiviral T cell responses enhances the protection by antibody-eliciting vaccines is however unclear. Here, we assessed this query in mouse CMV (MCMV) infection models by developing synthetic vaccines with humoral immunity potential, and deliberately adding antiviral CD8^+^ T cells. To induce antibodies against MCMV, we developed a DNA vaccine encoding either full-length, membrane bound glycoprotein B (gB) or a secreted variant lacking the transmembrane and intracellular domain (secreted (s)gB). Intradermal immunization with an increasing dose schedule of sgB and booster immunization provided robust viral-specific IgG responses and viral control. Combined vaccination of the sgB DNA vaccine with synthetic long peptides (SLP)-vaccines encoding MHC class I-restricted CMV epitopes, which elicit exclusively CD8^+^ T cell responses, significantly enhanced antiviral immunity. Thus, the combination of antibody and CD8^+^ T cell-eliciting vaccines provides a collaborative improvement of humoral and cellular immunity enabling enhanced protection against CMV.

## Introduction

Human cytomegalovirus (HCMV), a member of the β-herpesvirus family, is estimated to infect 60-80% of the world population. In healthy individuals, CMV establishes low-level viral persistence with little or no clinical symptoms with the exception of sporadically causing a mononucleosis-like illness (1). However, in immunocompromised individuals, including both solid organ and bone marrow-transplantation patients and HIV-infected persons, HCMV infection often causes serious complications. Moreover, congenital HCMV infection in the immunological immature unborn and newborn babies can cause severe morbidity, lifelong invalidity and even mortality (2). Although treatment options such as antiviral drugs and cellular therapy are available against HCMV-associated disease, preventive strategies such as vaccines are highly desired. Antiviral drugs require prolonged treatment, are accompanied by significant toxicity, and viral resistance to the drug is not uncommon (3). Despite ongoing efforts, no licensed effective prophylactic or therapeutic HCMV vaccines are available yet.

Infection with CMV results in activation of basically all arms of the immune system. In-depth studies documented that innate, humoral and cellular immune responses play important roles in the control of CMV infection and disease (2, 4). The impact on the immune system, however, is highly dependent on the infectious dose (5–7). The contribution of antibodies for protection against and control of CMV is mainly associated with restricting viral dissemination, limiting recurrent infection and the severity of the disease (8–10). Mothers that have HCMV antibodies before conception, transmit infection to the fetus at a lower frequency than women with primary infections (11), and passive immunization with HCMV antibodies can protect against congenital HCMV infection in newborns (12). Moreover, if antibodies specific to HCMV upon primary maternal infection are of low avidity and poor neutralizing activity, a higher transmission of viral infection from mother to fetus occurs (13). The administration of HCMV-specific antibodies to transplant recipients also results in reduction of HCMV-associated disease (14). The majority of the antibodies with virus-neutralizing capacity bind to the CMV glycoproteins, used for host cell entry. Especially virus-neutralizing antibodies against glycoprotein B (gB), a major envelope glycoprotein involved in cell attachment and penetration, accounts for the neutralizing antibody response to HCMV (15, 16). Moreover, also non-neutralizing anti-gB antibodies have been shown to exhibit protective capacity (17–19), which may be caused by induction of antibody-dependent cellular cytotoxicity (ADCC), antibody-dependent cellular phagocytosis (ADCP), and complement-dependent cytotoxicity (CDC) (20).

In addition to the humoral response, the T cell-mediated immune response is another major mechanism for controlling and restricting CMV replication in hosts (21). Functional CMV-specific CD8^+^ and CD4^+^ T cells are activated and expanded during primary infection. The ensuing T cell response is characterized by the maintenance of large oligoclonal T cell populations that remain high or even increase over time (22, 23). This phenomenon, named memory inflation (24), is not unique to CMV infection, but this virus seems to be most effective in triggering memory inflation (25). The CMV-specific memory CD8^+^ T cells have an advanced differentiated state and are able to lyse virus-infected cells and suppress intracellular virus replication by the secretion of IFN-γ and TNF (26). CD4^+^ T cells also have direct effects on viral replication by secretion of IFN-γ, in addition to supporting antibody and memory CD8^+^ T cell responses (27). Clinical data from transplant patients and HIV-infected individuals exposed a crucial role for CD8^+^ and CD4^+^ T cells in the control of HCMV (28–32). Moreover, the administration of CMV-specific CD8^+^ T cells by adoptive transfer limits CMV disease in experimental CMV settings and in the clinic (33–36). Especially, the strong inflationary CD8^+^ T cell responses against the immunogenic pp65 and IE1 CMV proteins are central in CMV control (37–39). In line with this, also the inflationary CD8^+^ T cells in murine models have strong antiviral capacity (40, 41).

Based on the success of numerous prophylactic vaccines designed to elicit antibodies, several vaccines aiming to induce protective humoral responses against HCMV were developed (42). The recombinant monomeric gB vaccine adjuvanted with MF59 was clinically tested and demonstrated a 50% efficacy in prevention of HCMV infection in CMV-seronegative women (43, 44). Since the protective capacity of the gB vaccines is considered to primarily depend on the induction of antibodies (45), a possible cause for the lack of higher efficacy could be the absence of strong CD8^+^ T cell responses by this vaccine. Accordingly, CMV vaccines were developed that induce both antibody and CD8^+^ T cell responses (reviewed in (42, 46)). However, it remained unclear whether addition of CD8^+^ T cell responses could actually aid antibody-mediated protection.

Previously, our group developed a synthetic long peptide (SLP) vaccine platform inducing robust and functional CMV-specific CD8^+^ T cell responses, resulting in reduced viral replication upon challenge (40). These SLP vaccines did not elicit MCMV-specific antibody or CD4^+^ T cell responses, indicating that vaccine-induced CD8^+^ T cells can operate solely to control viral infection. In this study, we aimed to demonstrate whether these vaccine-induced CD8^+^ T cell responses have an added value to antibody-eliciting vaccines. For this purpose, we developed DNA vaccines encoding gB, and subsequently analyzed the potency of a combinatorial DNA and SLP synthetic vaccine approach. We show that combined administration of a DNA vaccine eliciting a humoral response and an SLP vaccine eliciting antiviral CD8^+^ T cell responses results in more efficient control of lytic MCMV infection, which unequivocally demonstrates the need for directing both CMV-specific B and T cell immunity to combat CMV-associated disease.

## Results

### Booster Vaccination With DNA Vaccines Encoding Soluble gB Elicits Robust IgG Responses Against CMV

**Figure 1:**
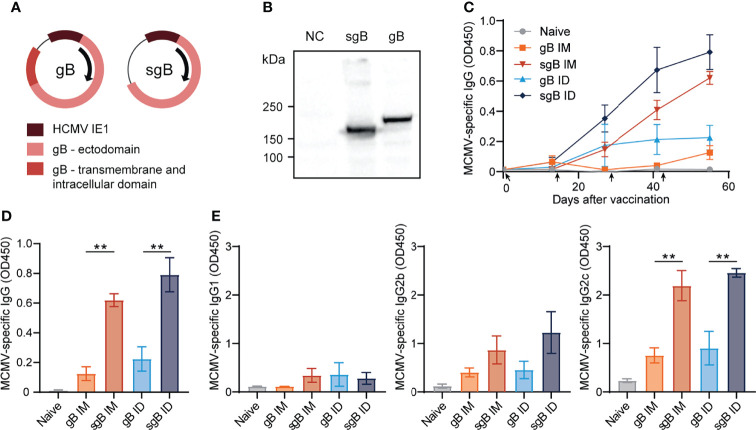
Booster vaccination with DNA vaccines encoding soluble gB elicits robust IgG responses against CMV. **(A)** Schematic representation of the DNA vaccines encoding gB or a soluble version of gB (sgB). **(B)** Western blot showing expression of sgB and gB proteins following transfection of the DNA vaccines into B16F10 cells. Negative control (NC) only received the transfection agents. **(C)** Kinetic analysis of the MCMV-specific IgG response in serum. Mice were vaccinated intradermally (ID) or intramuscularly (IM) four times at a two-week interval with 10 µg of sgB or gB DNA vaccine. At different time points blood was taken and serum was extracted to analyze the MCMV-specific IgG antibody response. Data shown are mean values ± SEM (n=4 per group). Arrows indicate vaccine injection time-points (day 0, 14, 28, 42). **(D)** Presence of MCMV-specific IgG antibodies for the different vaccinations two weeks after the fourth vaccination. Experiments were performed twice with similar outcome. **(E)** Presence of MCMV-specific IgG1, IgG2b and IgG2c subclasses for the different vaccinations two weeks after the fourth vaccination. Experiments were performed twice with similar outcome. One-way ANOVA was used for statistical analysis. **P<0.01.

To develop effective vaccines eliciting antibody-based protection against CMV, we constructed several DNA vaccines encoding glycoprotein B (gB). This glycoprotein is expressed on the surface of mouse and human CMV and is directly involved in viral entry into host cells (47, 48). To compare antibody responses against the full-length, membrane bound form of gB and a soluble, secreted form of gB (sgB, lacking the transmembrane and intracellular domain), two different DNA vaccines were tested (**Figures 1A, B**). The gB and sgB-encoding DNA vaccines were compared following administration in C57BL/6 mice *via* the intradermal (ID) and intramuscular (IM) route in a booster regimen. DNA vaccines encoding sgB administered either ID or IM resulted in an increase of the MCMV-specific IgG response upon booster vaccination, whereas the gB DNA vaccine elicited a lower IgG response that inferiorly responded to booster vaccination (**Figures 1C, D**). Moreover, vaccination *via* the ID route induced consistently a higher MCMV-specific IgG antibody response compared to IM vaccination, and this was observed after immunization with both the secreted and membrane bound forms of gB (**Figures 1C, D**). Evaluation of the IgG isotypes revealed that the IgG1 response was overall subordinate in all vaccination groups (**Figure 1E**), while IgG2b and most profoundly IgG2c (the C57BL/6 mice equivalent of IgG2a) levels where strongly induced by the sgB DNA vaccine compared to the gB DNA vaccine. Thus, booster vaccination *via* the ID route with sgB-encoding DNA vaccines results in superior MCMV-specific IgG antibody responses.

### Dose-Escalating DNA-Based Vaccination Improves Antibody Responses and Viral Protection

**Figure 2:**
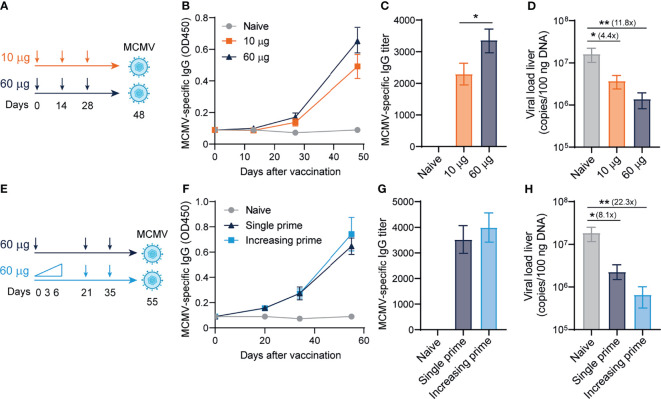
Dose-escalating DNA-based vaccination improves antibody responses and viral protection. **(A)** Vaccination schedule for mice were vaccinated intradermally three times with different doses (10 ug and 60 ug) of the sgB DNA vaccine. Three weeks after the final vaccination, mice were challenged intraperitoneally with 5 × 10^4^ PFU salivary gland-derived MCMV-Smith. **(B)** Kinetic analysis of the MCMV-specific IgG response in serum. Data shown are mean values ± SEM (n=8) **(C)** MCMV-specific endpoint binding IgG titers after three vaccinations. Data shown are mean values ± SEM (n=8). One-way ANOVA was used for statistical analysis. **(D)** At day 4 post-infection, livers were isolated and the viral genome copies were determined by PCR. The viral load is depicted as mean values ± SEM (n=8). **(E)** Vaccination schedule for mice vaccinated intradermally with either an increasing dose prime schedule (10 µg on day 0, 20 µg on day 3 and 30 µg on day 6) or a single prime dose of 60 µg on day 0, followed by two booster immunizations with a high dose (60 µg) of the sgB DNA vaccine. Three weeks after the final vaccination, mice were challenged intraperitoneally with 5 × 10^4^ PFU salivary gland-derived MCMV-Smith. **(F)** Kinetic analysis of the MCMV-specific IgG response in serum. Data shown are mean values ± SEM (n=8) **(G)** MCMV-specific endpoint binding IgG titers after two booster vaccinations are shown and represent mean values ± SEM (n=8). One-way ANOVA was used for statistical analysis. **(H)** At day 4 post-infection, livers were isolated and the viral genome copies were determined by PCR. The viral load is depicted as mean values ± SEM (n=8). Experiments were performed twice with similar outcome. A Kruskal-Wallis test was used for statistical analysis. *P<0.05, **P<0.01.

Next, we determined whether the antibody-eliciting sgB DNA vaccine could provide viral protection against MCMV challenge in a dose-dependent manner. C57BL/6 mice were ID vaccinated in a prime-boost-boost regimen with either 10 or 60 µg of the sgB DNA vaccine to measure a low and high dose of the sgB DNA vaccine (49), and 20 days after the last booster vaccination, mice were infected with MCMV (**Figure 2A**). The high-dose sgB vaccine induced a slightly higher MCMV-specific IgG response (**Figure 2B**) and IgG end-point titer (**Figure 2C**). Consistently, whereas the low-dose sgB DNA vaccine resulted in a 4-fold reduction of the viral load in the liver, the high-dose sgB vaccine lowered the viral load 12-fold compared to unvaccinated (naïve) mice (**Figure 2D**). These results show that low and especially high dosages of the sgB vaccine induces antibody responses able to provide protection against viral challenge.

Short-interval vaccination schedules with an increasing dose, mimicking the increment of foreign antigens as occurs upon natural infection, leads to improved vaccine-specific CD8^+^ T cell responses (50). To assess if such vaccine regimens could also improve the IgG response and associated viral protection of the sgB DNA vaccine, mice were immunized with increasing dosages (10, 20, 30 µg) at day 0, 3 and 6, respectively, or received a single dose vaccination. In both groups, the booster vaccines were provided as a single dose (**Figure 2E**). Dose escalation during prime did not significantly increase the MCMV-specific IgG response (**Figure 2F**) and the IgG end-point titer (**Figure 2G**). The increasing dosage priming effect nevertheless resulted in an increased reduction in the viral load in the liver upon viral challenge as compared to single dose priming (**Figure 2H**). Overall, these results show that using an increasing dose schedule with DNA vaccines during prime immunization improves protection against viral challenge.

### Combining the sgB DNA Vaccine With Synthetic Long Peptide Vaccines Enhances M38-Specific CD8^+^ T Cell Responses

**Figure 3:**
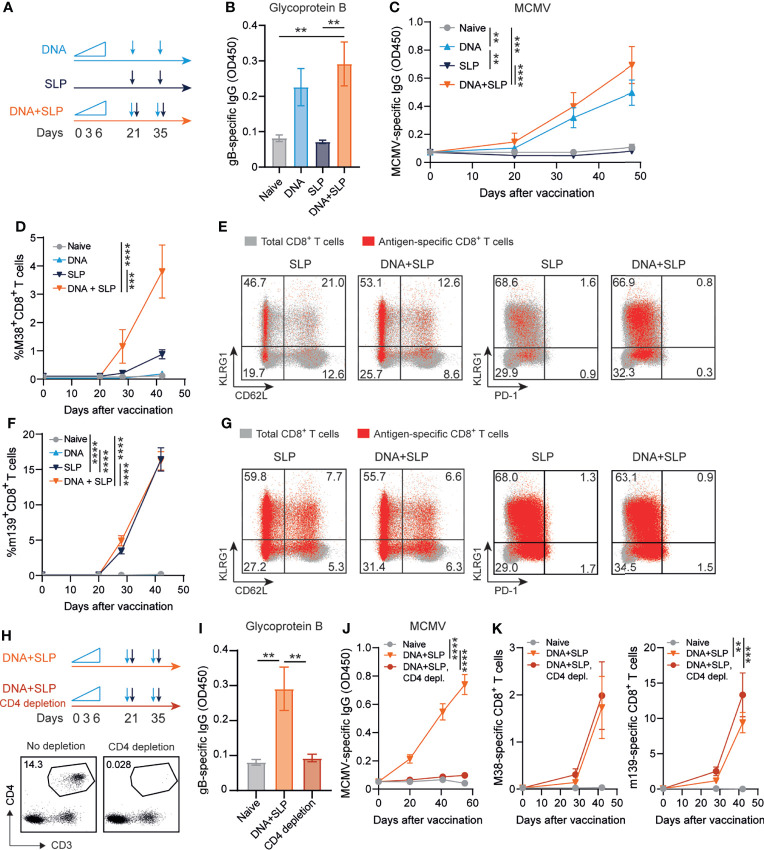
Combining the sgB DNA vaccine with synthetic long peptide vaccines enhances antibody and CD8^+^ T cell responses. **(A)** Vaccination schedule for mice vaccinated with the sgB DNA vaccine and/or the SLP vaccine. The sgB DNA vaccine was intradermally with an increasing dose prime schedule (10 µg on day 0, 20 µg on day 3 and 30 µg on day 6) followed by two booster immunizations with 60 µg. The SLP vaccine (synthetic long peptides containing the M38 and m139 class I epitopes, adjuvanted with CpG) was provided subcutaneously on day 21 and day 35. **(B)** Glycoprotein B-specific IgG response on day 72 after vaccination in blood. Data shown are mean values ± SEM (n=8). One-way ANOVA was used for statistical analysis. **(C)** Kinetics of the MCMV-specific IgG response in serum over time. Data shown are mean values ± SEM (n=8). One-way ANOVA on day 48 was used for statistical analysis. **(D, F)** Kinetic analysis of the M38- **(D)** and m139-specific **(F)** CD8^+^ T cell response. Data shown are mean values ± SEM (n=8). One-way ANOVA on day 48 was used for statistical analysis. **(E, F)** Representative flow cytometry plots showing the KLRG1 vs CD62L or KLRG1 vs PD-1 cell-surface expression on the total CD8^+^ T cells (gray) and M38- **(E)** and m139-specific **(G)** CD8^+^ T cells (red) in blood at day 7 post-boost vaccination. Indicated percentages are representative values for each group. Experiments were performed twice with similar outcome. **(H)** Vaccination schedule for mice vaccinated with the sgB DNA vaccine and the SLP vaccine with and without CD4^+^ T cell depletion during the vaccination period. The vaccines were administered as described in **(A)**. The CD4^+^ T cell depleting antibody was administered s.c. every six days. Representative flow cytometry plot showing confirmation of CD4^+^ T cell depletion in blood. **(I)** Glycoprotein B-specific IgG response on day 72 after vaccination in blood. Data shown are mean values ± SEM (n=9). One-way ANOVA was used for statistical analysis. **P<0.01. **(J)** Kinetics of the MCMV-specific IgG response in serum over time. Data shown are mean values ± SEM (n=9). One-way ANOVA on day 55 was used for statistical analysis. **(K)** Kinetic analysis of the M38- and m139-specific CD8^+^ T cell response. Data shown are mean values ± SEM (n=8). One-way ANOVA on day 42 was used for statistical analysis. **P<0.01, ***P<0.001, ****P<0.0001.

Previously, we showed that synthetic long peptide (SLP) vaccines eliciting exclusively MCMV-specific CD8^+^ T cell responses without the induction of antiviral CD4^+^ T cells or antibodies enhanced protection against viral challenge (40). To determine whether the induction of robust antiviral CD8^+^ T cell responses can improve antibody-mediated immunity, the antibody-eliciting sgB DNA vaccine was combined with the SLP vaccine eliciting CD8^+^ T cells against the MHC class I restricted epitopes in M38 and m139. Mice were vaccinated in a prime-boost regimen with either the sgB DNA vaccine, the SLP vaccine or the combination of both vaccines (**Figure 3A**).

As expected, the SLP vaccine did not elicit MCMV-specific antibodies but exclusively provoked CD8^+^ T cell responses while CD8^+^ T cell responses were not detected after sgB vaccination (**Figures 3B**–**D, F**). Moreover, antibodies against the SLPs were also not induced (**Supplementary Figure 1B**). Mice that received both the DNA and SLP-based vaccines inducing the antibody and CD8^+^ T cell responses, respectively, showed a similar gB-specific and MCMV-specific IgG response compared to mice that only received the DNA vaccine (**Figures 3B, C**). However, after prime SLP vaccination and after the booster, the combinatorial DNA/SLP vaccine improved the M38-specific CD8^+^ T cell response compared to the SLP vaccine alone (**Figure 3D**), whereas the m139-specific CD8^+^ T cell response was not affected, (**Figure 3F**). The combinatorial DNA/SLP vaccine induced a similar KLRG1^+^CD62L^-^ M38- and m139-specific CD8^+^ T cell population compared to the SLP vaccine alone after boost vaccination (**Figures 3E, G**). M38-specific and m139-specific CD8^+^ T cells induced upon either SLP or DNA/SLP vaccination did not upregulate the T cell exhaustion marker PD-1 (**Figures 3E, G**).

Next, we determined the role of CD4^+^ T cells for the development of the vaccine-specific B and T cell responses. First, we established whether the gB DNA and/or SLP vaccines vaccine elicited a CD4^+^ T cell response. Following sgB DNA vaccination, IFN-γ production of CD4^+^ T cells was clearly detected after stimulation with peptides spanning the MCMV gB protein, whereas stimulation with the SLPs did not elicit reactivity (**Supplementary Figure 1C**). Moreover, SLP vaccination did not elicit IFN-γ production by the CD4^+^ T cells after stimulation with gB protein or SLPs. To determine whether CD4^+^ T cell help is critical for the vaccine-specific B and T cell responses, we depleted the CD4^+^ T cells during the vaccination period (**Figure 3H**). CD4^+^ T cell depletion resulted in the absence of gB-specific and MCMV-specific IgG antibodies (**Figures 3I, J**). Depletion of the CD4^+^ T cells did however not affect the height of the M38- and m139-specific CD8^+^ T cell response (**Figure 3K**). Together, these results indicate that combining the sgB DNA vaccine with SLP vaccines results in similar levels of vaccines-specific antibody levels in a CD4^+^ T cell dependent manner, and enhancement of the M38-specific CD8^+^ T cell response.

### The Combination of sgB DNA Vaccines With CD8^+^ T Cell-Eliciting Synthetic Long Peptide Vaccines Is Superior in Protection Against MCMV

**Figure 4:**
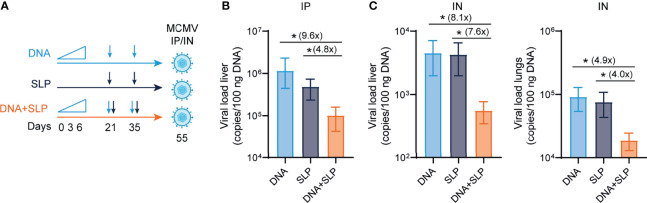
The combination of sgB DNA vaccines with the CD8^+^ T cell eliciting synthetic long peptide vaccines is superior in protection against MCMV. **(A)** Vaccination schedule for mice vaccinated with the sgB DNA vaccine and/or the SLP vaccine. The sgB DNA vaccine was intradermally with an increasing dose prime schedule (10 µg on day 0, 20 µg on day 3 and 30 µg on day 6) followed by two booster immunizations with 60 µg. The SLP vaccine (synthetic long peptides containing the M38 and m139 class I epitopes, adjuvanted with CpG) was provided subcutaneously on day 21 and day 35. Three weeks after the final vaccination, mice were challenged either intraperitoneally or intranasally with 5 × 10^4^ PFU salivary gland-derived MCMV-Smith. **(B)** At day 5 post intraperitoneal infection, livers were isolated. The viral genome copies were determined by PCR. The viral load is depicted as mean values ± SEM (n=8). **(C)** At day 5 post intranasal infection, livers and lungs were isolated. The viral genome copies were determined by PCR. The viral load is depicted as mean values ± SEM are shown (n=8). Experiments was performed twice with similar outcome. A Kruskal-Wallis test was used for statistical analysis. *P<0.05.

To determine the protective capacity of the combinatorial DNA/SLP vaccine, mice were challenged with MCMV *via* the intraperitoneal route, to mimic systemic infection that can occur upon organ transplantation of a CMV-positive donor into a CMV-negative recipient, or *via* the intranasal route, representing the natural route of CMV infection (51) (**Figure 4A**). The viral load in the liver after intraperitoneal challenge was 5-fold lower in the mice that received combinatorial vaccination as compared to single SLP vaccination, and 16-fold lower compared to single DNA vaccination (**Figure 4B**). Upon intranasal challenge, the combinatorial vaccine reduced the viral load in the liver 8-fold compared to SLP vaccination, and also 8-fold compared to DNA vaccination (**Figure 4C**). The protective capacity of the combinatorial SLP/DNA vaccine was also observed in the lungs of intranasally challenged mice. Here, the viral load of the combinatorial vaccine was 4 and 5-fold lower compared to single SLP and single DNA vaccination, respectively (**Figure 4C**).

Taken together, we show that the combinatorial DNA/SLP vaccine improves protection in different organs, compared to the single-arm vaccination strategies, indicating a synergistic effect of combining these vaccine platforms to enhance humoral as well as cellular responses against CMV.

## Discussion

Here we show in experimental CMV models that the effectivity of antibody-eliciting DNA vaccines against CMV infection *via* the intranasal and intraperitoneal route can be improved by the addition of CD8^+^ T cell responses induced by SLP vaccines. This experimental study allowed the direct comparison of vaccine platforms inducing either a CMV-specific antibody or a CD8^+^ T cell response or the combination thereof, and emphasized the importance of inducing both the humoral and cellular immune response to counteract CMV-associated disease. In this respect, vaccines such as V160, a conditionally replication-defective vaccine derived from the AD169 strain, are of interest because of the induction of both neutralizing antibody titers and cellular responses (52, 53).

The sgB antibody-eliciting DNA vaccine outperformed the gB DNA vaccine, which is in line with Lauterbach et al., who showed >10 fold higher IgG titers after DNA vaccines encoding soluble antigen compared to membrane bound antigen (54). This may be explained by trapping of sgB by follicular dendritic cells and subsequent presentation to B cells (55). Further optimization of the sgB antibody-eliciting DNA vaccine and CD8^+^ T cell-eliciting SLP vaccine could be achieved by several possibilities. First, although gB vaccines elicit CD4^+^ T cell responses (56, 57) (**Supplementary Figure 1C**) stronger induction of CMV-specific CD4^+^ T cell responses with SLP vaccines for example has direct antiviral effects (58), and may facilitate both the CD8^+^ T cell and antibody response (59). With respect to the latter, the sgB vaccine may already induce sufficient (selective) CD4^+^ T cell help to facilitate the IgG response. The CD4^+^ T cell help mediated by the gB vaccine is in our setting, however, not instrumental in the increment of the M38-specific CD8^+^ T cell response after DNA/SLP booster vaccination. This elevation may be caused by an adjuvant effect of the DNA vaccine, which is coupled to immunostimulatory DNA sequences like unmethylated CpG motifs and cytoplasmic DNA sensors in the STING/IRF7 pathway (60, 61). In contrast to CD4^+^ T cell responses, CD8^+^ T cell responses, are not elicited by gB vaccines (57), which is in line with studies demonstrating the absence of immunodominant class I-restricted epitopes in the gB protein (62, 63). Moreover, the addition of an agonistic antibody to OX40, a costimulatory receptor on activated T cells, could be added to enhance CD4^+^ and CD8^+^ T cell-based protection (58). Furthermore, vaccines using the trimeric form of gB as an antigen instead of monomeric resulted in 50-fold times higher neutralizing titers (64), and neutralizing antibodies against the trimeric (gH/gL/gO) and pentameric complex (gH/gL/UL128/UL130/UL131A) have shown neutralizing activity and prevention of infection of epithelial, endothelial cells and fibroblasts (65–68). Thus, further enhancing antibody-mediated protection by optimizing gB-based vaccines and adding other immunogenic proteins are both advisable. Designing one vaccine platform inducing both strong antibody and T cell responses against CMV may, however, be challenging but combined formulations as used here may be an option for further development of synthetic vaccines.

Taken together, the findings here establish that deliberate induction of humoral and cellular immunity enables enhanced protection against herpesvirus infection. Especially, antibody-mediated protection can become more effective by addition of strong vaccine-induced CD8^+^ T cell responses, thereby highlighting the importance of designing CMV vaccines that elicit both strong T and B cell responses.

## Materials and Methods

### Mice

C57BL/6 mice were purchased from Janvier (Le Genest-Saint-Isle, France). Mice were maintained under specific-pathogen-free conditions at the Central Animal Facility of Leiden University Medical Center (LUMC). Mice were aged 8-10 weeks at the start of each experiment. All animal experiments were approved by the Animal Experiments Committee of the LUMC and performed according to the Dutch Experiments on Animals Act that serves as the implementation of the guidelines on the protection of experimental animals by the Council of Europe.

### DNA Construct, Peptides and Vaccination

Full-length glycoprotein B (gB, NCBI Gene symbol MuHV1_gp059) was amplified by PCR from a BAC clone containing the Murid betaherpesvirus 1 genome (MCMV K181, pSM3fr-MCK-2fl). As forward primer, 5’-CCAAGCTGTCTAGAGCCGCCACC ATG GCA AGA AGA AAC GAA AGA GGA TGT C-3’ containing a single Ser-to-Ala substitution at position 2 was used to introduce a consensus Kozak sequence. A reverse primer 5’- GT TTA CTT CTC GAA CTG AGG GTG AGA CCA AGC GCT GTA CTC GAA ATC GGA GTC CTC C-3’ was used containing a Strep-tag (underlined, amino acid sequence SA-WSHPQFEK) and stop codon. To amplify a gene fragment encoding secreted glycoprotein (sgB), the same forward primer was used in combination with the reverse primer 5’- GT TTA CTT CTC GAA CTG AGG GTG AGA CCA AGC GCT AAA CGG GTT CGT CAG GAA GC-3’ to generate a construct replacing the transmembrane and cytoplasmic domains (aa 787 to 937) with a Strep-tag. Both PCR fragments were assembled into a pVAX-based expression vector containing a HCMV IE1-promoter, a rabbit beta-globin poly-A signal and kanamycin resistance marker using the NEBuilder HiFi DNA Assembly kit (New England Biolabs, Ipswich, MA, USA). Plasmids were propagated in E. coli cultures and purified using Nucleobond Xtra maxi EF columns (Macherey-Nagel) according to manufacturer’s instructions. DNA constructs were verified by double-stranded Sanger sequencing (Baseclear). For vaccination, plasmids were column-purified twice, each time using a fresh column, and dissolved at 3 mg/ml in Tris : EDTA buffer (1:0.1 mM). Mice were intradermally or intramuscularly vaccinated with 30 µL DNA-lipid nanoparticles containing cationic lipid SAINT-18 (provided by Synvolux Therapeutics) in a 1: 0.75 ratio (µg DNA: nmole SAINT-18) in 0.9% NaCl at the tail base.

Synthetic long peptides (SLP) containing MHC class I-restricted T cell epitopes from MCMV proteins M38 and m139 (M38_316-323_ and m139_419-426_) (40) were synthesized at the peptide facility of the LUMC. Mice were vaccinated subcutaneously at the tail base with a mixture of 50 µg of each SLP and 20 µg CpG (ODN 1826, *Invivogen*) in 50 µL PBS.

### Western Blot Analysis

Expression of DNA vaccines was verified *in vitro* by transfection and Western blotting using a gB-specific antibody. Briefly, mouse B16F10 cells were seeded in a 6-wells plate and transfected the next day with 1 µg DNA and Saint-DNA transfection reagent (Synvolux Therapeutics) according to the manufacturer’s instructions. After two days, cells were washed in PBS, and lysed in Laemmli buffer containing beta-mercaptoethanol. Equal amounts of total cell lysates were separated on a 10% polyacrylamide gel and electrophoretically transferred to nitrocellulose membranes (Amersham Protran). Membranes where then incubated with a primary antibody specific for MCMV glycoprotein B (cat. MCBG11, Alpha Diagnostic Intl.) and HRP-conjugated swine anti-rabbit secondary antibody (Dako Agilent). Antibody binding was visualized by chemiluminescence using Clarity Western ECL substrate (Bio-Rad).

### MCMV Preparation, Infection and Determination of Viral Load

MCMV-Smith was obtained from the American Type Culture Collection (ATCC VR-194; Manassas, VA, USA) and virus stocks were prepared from salivary glands of infected BALB/c mice. The viral titers of the produced virus stocks were determined by viral plaque assays with 3T3 mouse embryonic fibroblasts (MEFs) (ATCC). Age- and sex-matched mice were immunized intraperitoneally with 1 × 10^4^ PFU MCMV. 20 days upon the last booster vaccination, mice were challenged with 5 × 10^4^ PFU MCMV. At day 5 post MCMV challenge, viral loads in liver and lungs were determined by real-time PCR as described previously (5).

### Serum Antibody Detection by ELISA

Blood of mice was collected *via* the tail vein. Serum was collected upon centrifugation and stored at -20°C until further use. MCMV-specific IgG levels were measured by ELISA as described before (5), and endpoint binding antibody titers were determined by calculating the dilution at which the OD450 was twice as high as the background of the assay. In brief, 96-well plates (Nunc MaxiSorp) were coated overnight at 4°C with tissue culture derived MCMV-Smith in bicarbonate buffer (pH 9.6) and washed with PBS. After blocking the plates for 1 h at 37°C with blocking buffer (PBS/5% milk powder) and washing with PBS containing 0.05% Tween, diluted sera (in PBS/1% milk powder) were added and incubated for 1h at 37°C. For the gB-specific IgG levels, 96-well plates (Nunc MaxiSorp) were coated overnight at 4°C with recombinant glycoprotein B (1 µg/mL, Alpha Diagnostic International) in bicarbonate buffer (pH 9.6), washed with PBS/0.05% Tween and blocked with PBS containing 1% bovine serum albumin (BSA) and 0.05% Tween (blocking buffer) for 1 h at room temperature. Plates were washed with PBS/0.05% Tween and incubated with serial dilutions of mouse sera in blocking buffer and incubated for 1 h at room temperature. The M38- and m139-specific IgG levels were determined by coating streptavidin coated plates (Kaivogen) with 5 µg/mL biotinylated M38 and m139 SLP in bicarbonate buffer (pH 9.6) and washed with PBS/0.05% Tween and blocked with PBS/5% milk powder for 1 h at room temperature. Plates were washed with PBS/0.05% Tween and incubated with serial dilutions of mouse sera in PBS/1%BSA and incubated for 1 h at room temperature. For all ELISAs, plates were then washed with PBS/0.05% Tween, after which horseradish peroxidase (HRP)-conjugated IgG (diluted in PBS/1% milk powder was added) was incubated for 1 h at 37°C. To develop the plates, 50 µL of TMB 3,3=,5,5=tetramethylbenzidine) (Sigma-Aldrich) was added to each well and incubated for 10 minutes at room temperature. Plates were measured with a microplate reader (model 680; Bio-Rad) at 450 nm within 5 minutes after the reaction was stopped by the addition of 50 µL 1M H_2_SO_4_.

### Flow Cytometry

Blood was collected *via* the tail vein and antigen-specific CD8^+^ T cell responses in blood were evaluated with cell surface staining, performed as previously described (69). In brief, upon lysis of erythrocytes single-cell suspensions were incubated with fluorescently-labeled antibodies and MHC class I tetramers for 30 minutes at 4˚C. MHC class I tetramers specific for M38_316–323_ and m139_419–426_ MCMV epitopes were used to stain M38-and m139-specific CD8^+^ T cells. Fluorochrome-conjugated antibodies specific for mouse CD3, CD4, CD8, CD62L, KLRG1, IFN-γ, and PD-1 were purchased from Biolegend or eBioscience. Dead cells were excluded with the use of 7-aminoactinomycinD (7-AAD) (Invitrogen). For examination of intracellular IFN-γ production, white blood cells were stimulated with long peptides for 8 h of which the last 6 h in presence of brefeldin A (Golgiplug; BD Pharmingen). Flow cytometric acquisition was performed on a BD Fortessa flow cytometer (BD Biosciences) or Aurora Cytek spectral analyzer, and samples were analyzed using FlowJo software (TreeStar).

Flow cytometric acquisition was performed on a LSR Fortessa cytometer (BD Biosciences) and samples were analyzed using FlowJo software (TreeStar).

### CD4^+^ T Cell Depletion

CD4^+^ T cell depleting monoclonal antibodies (clone GK1.5, BioXcell) were administered intraperitoneally twice per week, starting 4 days before vaccination. For the first injection, mice received 150 µg per mouse, and CD4^+^ T cell depletion was maintained with 50 µg per mouse.

### Statistical Analysis

Significance between groups was evaluated by performing an unpaired Student’s T test or ANOVA. To evaluate statistical significant difference of the viral load, a Kruskal-Wallis test was used. All statistical analyses were performed in Prism (Graphpad software). The level of statistical significance was set at P<0.05.

## Data Availability Statement

The original contributions presented in the study are included in the article/**Supplementary Material**. Further inquiries can be directed to the corresponding author.

## Ethics Statement

The animal study was reviewed and approved by Animal Experiments Committee of the LUMC.

## Author Contributions

IP designed and performed most of the experiments and data analysis, and wrote the manuscript. SD, DV, DB and AR assisted in experiments and reviewed the manuscript. WH and GZ designed and assisted in experiments and reviewed the manuscript. JB and FO provided conceptual input and reviewed the manuscript. RA designed and supervised the study, wrote the manuscript and provided funding for the study. All authors contributed to the article and approved the submitted version.

## Funding

This work was funded by an Applied and Engineering Sciences (TTW) grant from the Dutch Research Council (NWO, grant number 15830 (awarded to RA).

## Conflict of Interest

WH, JvB, and GZ are employees of Immunetune BV.

The remaining authors declare that the research was conducted in the absence of any commercial or financial relationships that could be construed as a potential conflict of interest.

## Publisher’s Note

All claims expressed in this article are solely those of the authors and do not necessarily represent those of their affiliated organizations, or those of the publisher, the editors and the reviewers. Any product that may be evaluated in this article, or claim that may be made by its manufacturer, is not guaranteed or endorsed by the publisher.

## Supplementary Material

Supplementary Figure 1 **(A)** Representative flow cytometry gating of tetramer-positive CD8^+^ T cells. **(B)** M38- and m139-specific IgG antibody response on day 72 upon vaccination. **(C)** Intracellular IFN-γ production of CD4^+^ T cells in blood at day 79 after DNA, SLP or combined DNA/SLP vaccination. Blood single cell suspension was stimulated with the M38 SLP, m139 SLP, synthetic long peptides spanning the whole MCMV gB protein no peptide.
