# Supplementary figures and images for: Dominant Antiviral CD8+ T Cell Responses Empower Prophylactic Antibody-Eliciting Vaccines Against Cytomegalovirus
Source: Front Immunol. 2022 Jan 27;13:680559. doi: 10.3389/fimmu.2022.680559 (PMC8828907; doi:10.3389/fimmu.2022.680559)

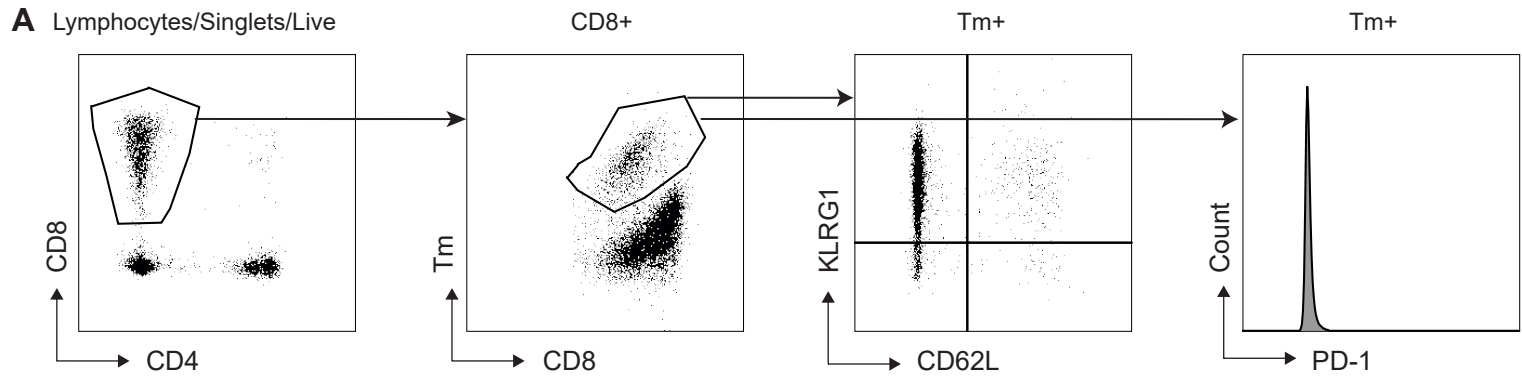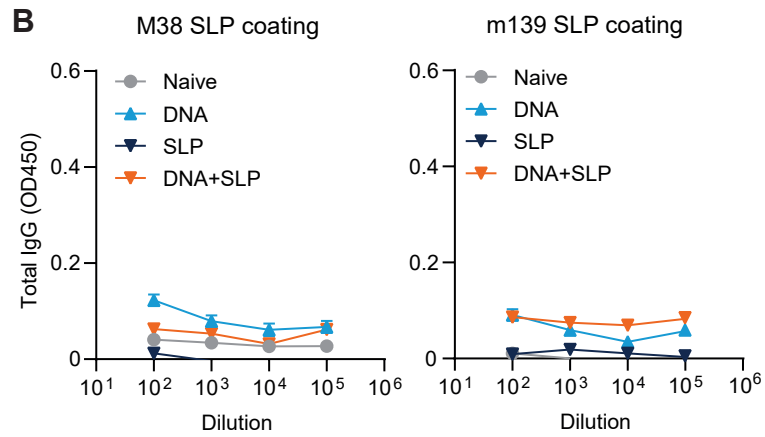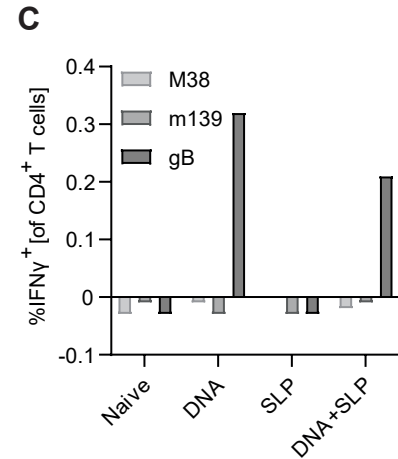

Supplement: Supplementary Figure 1 — (A) Representative flow cytometry gating of tetramer-positive CD8+ T cells. (B) M38- and m139-specific IgG antibody response on day 72 upon vaccination. (C) Intracellular IFN-γ production of CD4+ T cells in blood at day 79 after DNA, SLP or combined DNA/SLP vaccination. Blood single cell suspension was stimulated with the M38 SLP, m139 SLP, synthetic long peptides spanning the whole MCMV gB protein no peptide. [file DataSheet_1.pdf]
